# Predictive value of interim positron emission tomography in diffuse large B-cell lymphoma: a systematic review and meta-analysis

**DOI:** 10.1007/s00259-018-4103-3

**Published:** 2018-08-23

**Authors:** Coreline N. Burggraaff, Antoinette de Jong, Otto S. Hoekstra, Nikie J. Hoetjes, Rutger A. J. Nievelstein, Elise P. Jansma, Martijn W. Heymans, Henrica C. W. de Vet, Josée M. Zijlstra

**Affiliations:** 1 Department of Hematology, VU University Medical Center, Cancer Center, De Boelelaan 1117, 1081 HV Amsterdam, The Netherlands; 2 Department of Radiology and Nuclear Medicine, University Medical Center Utrecht, Utrecht, The Netherlands; 3 Department of Radiology and Nuclear Medicine, Amsterdam UMC, Vrije Universiteit Amsterdam, Cancer Center Amsterdam, Amsterdam, The Netherlands; 4 Department of Epidemiology and Biostatistics, Amsterdam UMC, Vrije Universiteit Amsterdam, Amsterdam Public Health Research Institute, Amsterdam, The Netherlands

**Keywords:** Aggressive non-Hodgkin’s lymphoma, Diffuse large B-cell lymphoma, Positron-emission tomography, Systematic review, Meta-analysis

## Abstract

**Purpose:**

Diffuse large B-cell lymphoma (DLBCL) represents the most common subtype of non-Hodgkin lymphoma. Most relapses occur in the first 2 years after diagnosis. Early response assessment with ^18^F-fluoro-2-deoxy-D-glucose (^18^F-FDG) positron emission tomography (PET) may facilitate early change of treatment, thereby preventing ineffective treatment and unnecessary side effects. We aimed to assess the predictive value of visually-assessed interim ^18^F-FDG PET on progression-free survival (PFS) or event-free survival (EFS) in DLBCL patients treated with first-line immuno-chemotherapy regimens.

**Methods:**

For this systematic review and meta-analysis Pubmed, Embase, and the Cochrane Library were searched until July 11, 2017. Prospective and retrospective studies investigating qualitative interim PET response assessment without treatment adaptation based on the interim PET result were eligible. The primary outcome was two-year PFS or EFS. Prognostic and diagnostic measures were extracted and analysed with pooled hazard ratios and Hierarchical Summary Receiver Operator Characteristic Curves, respectively. Meta-regression was used to study covariate effects.

**Results:**

The pooled hazard ratio for 18 studies comprising 2,255 patients was 3.13 (95%CI 2.52–3.89) with a 95% prediction interval of 1.68–5.83. In 19 studies with 2,366 patients, the negative predictive value for progression generally exceeded 80% (64–95), but sensitivity (33–87), specificity (49–94), and positive predictive values (20–74) ranged widely.

**Conclusions:**

These findings showed that interim ^18^F-FDG PET has predictive value in DLBCL patients. However, (subgroup) analyses were limited by lack of information and small sample sizes. Some diagnostic test characteristics were not satisfactory, especially the positive predictive value should be improved, before a successful risk stratified treatment approach can be implemented in clinical practice.

**Electronic supplementary material:**

The online version of this article (10.1007/s00259-018-4103-3) contains supplementary material, which is available to authorized users.

## Introduction

Diffuse large B-cell lymphoma (DLBCL) represents the most common subtype of adult non-Hodgkin lymphoma (NHL) cases, and is associated with an aggressive clinical course. There are several potentially effective first-line chemotherapy regimens of which most consist of cyclophosphamide, doxorubicin, vincristine, and prednisone (CHOP). The addition of the monoclonal antibody rituximab (R) to this regimen (R-CHOP) has significantly improved the outcome of DLBCL patients [1, 2]. However, treatment failure is still an important problem as the 3-year progression-free survival (PFS) of DLBCL patients is approximately 60–70% [3].

Commonly used prognostic indices are the International Prognostic Index (IPI) [4, 5], or the more powerful Revised-IPI (R-IPI) [6], and National Comprehensive Cancer Network IPI (NCCN-IPI) [7]. These indices can be used for risk-stratification to predict a poor outcome after R-CHOP. It is important to identify a poor outcome as soon as possible because these patients could benefit from a switch to a second-line treatment or high-dose chemotherapy (HDCT) with autologous stem cell transplantation (ASCT) as an upfront treatment [8]. ^18^F-fluoro-2-deoxy-D-glucose (^18^F-FDG) positron emission tomography (PET) after a few cycles of therapy, also known as interim ^18^F-FDG PET, is of increasing interest, as it may facilitate early change of treatment and prevent unnecessary side effects [9]. In recent decades several visual criteria for interpretation of ^18^F-FDG PET have been developed, for example, the EORTC, PERCIST, and International Harmonization Project (IHP) criteria as well as the Deauville scoring system [9–13]. Nowadays the latter is widely adopted for interpretation of response evaluation with ^18^F-FDG PET in DLBCL [9, 13].

Interim ^18^F-FDG PET has shown high predictive value in Hodgkin lymphoma [14]; however, according to previous reviews, the role of interim ^18^F-FDG PET in DLBCL is still unknown [15–18]. From these studies it can be concluded that heterogeneity in patient populations, therapy regimens, PET scanners, timing of the interim ^18^F-FDG PET scans, and/or differences in the visual criteria used for interpretation of the interim ^18^F-FDG PET scans made it hard to clarify the accuracy of interim ^18^F-FDG PET to predict clinical outcome in DLBCL.

Therefore, we performed a new systematic review and meta-analysis, focusing on DLBCL patients only, assessing both the hazard ratio (HR) and diagnostic parameters (sensitivity, specificity, and predictive values) of interim ^18^F-FDG PET on PFS or event-free survival (EFS) in patients with DLBCL treated with first-line immuno-chemotherapy regimens. The primary outcome measure was PFS (preferably) or EFS at 2 years, since DLBCL patients who are event-free after 24 months have demonstrated an overall survival (OS) comparable to an age- and sex-matched general population [19]. In order to reduce the previously described heterogeneity we performed several subgroup analyses, for example, by the type of ^18^F-FDG PET scanner and the type of visual criteria used for interpretation of the interim ^18^F-FDG PET scans. In this meticulously performed review we contacted the authors for additional information if necessary.

## Materials and methods

### Search strategy

For this systematic review and meta-analysis we searched in collaboration with a medical librarian Pubmed/MEDLINE, Embase, and the Cochrane Library databases from onset until July 11, 2017 with a language restriction to English, French, Dutch, or German. Our search strategy contained a combination of various indexed terms and free text words for “positron emission tomography” and “non-Hodgkin lymphoma” (full search strategy Supplemental Table 1). We included full-text publications of original prospective and retrospective studies. Excluded were conference abstracts, letters, comments, editorials, review articles, animal studies, and case reports. Reference lists of included articles were checked to identify additional eligible studies.

### Study selection: Eligibility criteria

#### Patients

Adult patients treated with first-line immuno-chemotherapy regimens for stage I-IV DLBCL were considered as our target population. We excluded studies that investigated HIV-related lymphoma, central nervous system (CNS) lymphoma involvement, or post-transplant lymphoproliferative disease (PTLD). Studies containing less than 80% of DLBCL subtype were excluded, unless subgroup data for DLBCL were presented or if the remaining 20% had PMBCL or FL grade 3B [20]. Studies including ten patients or less were classified as case series and therefore also excluded.

#### Treatment procedures

Studies in which a change of treatment was based on the interim ^18^F-FDG PET result and prospective PET-adapted trials were not included. However, we allowed a change of therapy in patients with clinical evidence of progressive disease during first-line treatment [9].

We included all R-CHOP-like treatments as first-line treatment strategies [1, 2, 21–23], but we excluded studies if ≤50% of patients received rituximab. Therapies using other (new generation) monoclonal antibodies were excluded.

Studies with autologous stem cell transplantation (ASCT) were eligible if this strategy was part of the preplanned first-line treatment. Radiotherapy was accepted if the decision to give radiotherapy was preplanned or used for consolidation of PET positive sites at the end of first-line treatment, but not affected by interim ^18^F-FDG PET results. If studies did not report on the use of ASCT or radiotherapy, we assumed that no ASCT or radiotherapy was given based on interim ^18^F-FDG PET result.

#### Interim ^18^F-FDG PET procedures

An interim ^18^F-FDG PET scan should have been performed after the first, second, third, or fourth treatment cycle. PET only as well as PET/CT systems were considered eligible. Use of other radiopharmaceuticals than ^18^F-FDG were not accepted.

We focused on visual interpretation criteria only, as nowadays, semi-quantitative PET strategies are used for research purposes only and are not standard in the current guidelines yet [13]. PET response criteria were grouped into three categories: Deauville score (DS) on a 5-point scale [9, 13], International Harmonization Project (IHP) [12], and custom visual criteria (i.e. not based on consensus guidelines).

#### Outcome measures

The primary outcome measure was defined as PFS (preferably) or EFS at 2 years. We included studies with a minimum median follow-up period of 24 months in surviving patients (or for the entire study population), because most patients experience relapse or progression of their disease in the first 2 years after their diagnosis [24, 25].

### Data extraction and quality assessment

After removing duplicates, two authors independently screened titles and abstracts of the search results for eligibility (CNB and NH, AdJ, or HCWdV). The decision to include studies in the review was based on the full-text articles (CNB and AdJ or HCWdV). Extensive data extraction forms (available upon request) were developed which included the criteria from the methodological checklists for diagnostic accuracy studies (QUADAS-2) [26] and for prognostic studies (QUIPS) [27]. The forms were tested in a few articles and used independently by two review authors (CNB, AdJ). Consensus meetings (with three experts in nuclear medicine, hematology, and methodology, respectively) were organized to solve disagreements and to decide on eligibility of the final study selection. Besides general information about study design, patients, treatment, interim ^18^F-FDG PET performance, and outcome measures (used for qualitative study descriptions and determination of eligibility) we extracted outcomes on two types of predictive parameters.

For the first predictive meta-analysis we extracted univariate hazard ratios (HRs) and their corresponding 95% confidence intervals. If this data was not reported and not provided after contacting the authors, we used the methods of Tierney et al. [28] to deduce these from reported parameters or from the Kaplan-Meier (KM) curves, using numbers at risk when available.

For the second predictive meta-analysis we used a diagnostic approach and constructed 2 × 2 contingency tables to calculate sensitivity, specificity, positive predictive value (PPV), and negative predictive value (NPV) of interim ^18^F-FDG PET for prediction of two-year PFS and - EFS. If no two-year survival percentages were reported we estimated the percentages from the KM curves at this time-point. If information was missing or unclear authors were contacted. A maximum of three reminders were sent. In case of no reply we used the information that was available from the original publication. Individual patient data was not requested for this meta-analysis.

### Statistical analyses

#### Two approaches of meta-analysis

For the meta-analyses of the HRs, individual log hazard ratios (HRs) and standard errors (SE) were pooled using a random-effects model (REML, restricted maximum likelihood). Together with the individual study results, the pooled effect estimate—expressed as HR and 95% confidence interval—was visualized in a Forest plot. Between-study heterogeneity was assessed by using Cochran’s Q and I^2^ statistics [29]. A 95% prediction interval around the HR was calculated to predict the expected range of the HR of a new (future) study [30]. A funnel plot was presented to visually assess if publication bias was likely [31].

For the diagnostic meta-analysis, the pooled sensitivity and specificity was obtained by Hierarchical Summary ROC curve (HSROC) models and ROC curves constructed in RevMan [32] using the input parameters of the HSROC models*.*

#### Influence of covariates

Several prespecified subgroup analyses—which included both clinical and methodological issues—were performed using univariate meta-regression models for the HRs and as covariate interaction term in the HSROC models. The following subgroup analyses were performed: study design (retrospective or prospective studies; blinded review or not reported; PFS or EFS), characteristics of patients (100% DLBCL or between 80 and 100%), treatments (ASCT upfront or not, preplanned or consolidative radiotherapy used or unknown), properties of scans (PET/CT or a combination of PET/CT and PET standalone systems, availability of a baseline PET or CT), and scoring issues (DS -, IHP -, or custom criteria, central review or local review).

#### Software

Statistical analysis was performed in R (version 3.2.5) [33] using the Metafor package and SAS Proc Nlmixed was used for the HSROC models. A *P* value of less than 0.05 was considered statistically significant.

## Results

**Fig. 1:**
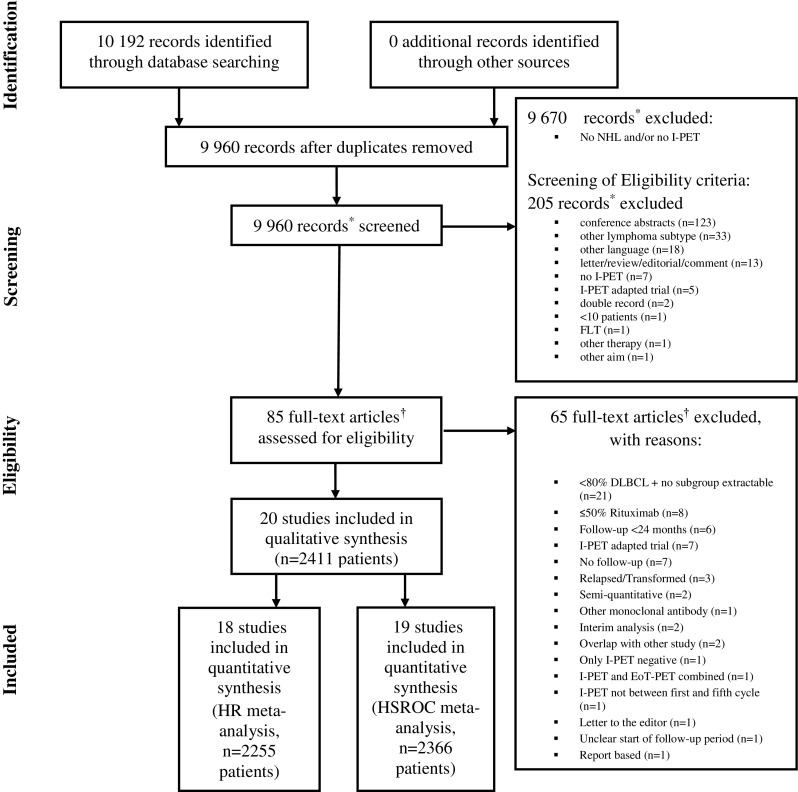
PRISMA flow diagram. ^*^Records refer to the title and abstract screening of the search results. ^†^Full-text articles refer to the full-text assessment of the selected articles from the title and abstract screening phase. **Abbreviations:** I-PET = interim ^18^F-FDG positron emission tomography, FLT = Fluorothymidine, DLBCL = diffuse large B-cell lymphoma, EoT-PET = end-of-treatment ^18^F-FDG positron emission tomography, HR = hazard ratio, HSROC = hierarchical summary receiver operating curve

The search yielded 9,960 records after removing duplicates; 290 concerned studies on NHL and interim FDG-PET, the other 9,670 records were excluded because they did not report on NHL or I-PET. 85/290 were potentially eligible and full-text articles were retrieved. After checking detailed inclusion and exclusion criteria we included 20 eligible studies in the qualitative systematic review; 19 out of 20 were eligible for the HRs evaluations and 18 out of 20 for the HSROC analyses (Fig. 1).

A total of 2,411 newly diagnosed DLBCL patients from 20 studies were assessed for this analysis. Table 1 shows the main study-, patient-, and treatment characteristics of the included studies. The number of included patients per study ranged from 32 to 327 (median 112, interquartile range 70–142). Seven studies had a prospective study design. The median age of the patients ranged from 54 to 65 years, with the exception of one study with a median age of 46 [40], and 45–67% of the patients were of male gender. Most studies included patients with Ann Arbor stage I/II as well as stage III/IV; in two studies less than 50% of the patients had stage III or IV [37, 45] and one study included patients with stage III and IV only [51]. First-line treatment regimens varied between and within the studies, but R-CHOP was the basic principle in all studies. Radiotherapy was given in most of the studies to selected patients (preplanned, e.g. in case of bulky disease or as a consolidation for residual lymphoma sites after treatment). Autologous stem cell transplantation had been planned upfront in three studies [44, 48, 50].

**Table 1:** Study- and patient characteristics

Study characteristics		Patient characteristics					Treatment characteristics		
First author, year	Study design	No. of patients	DLBCL	Age median (range)	Male	Stage III-IV	First-line treatment	RT	ASCT
Fan et al. (2017) [34]	Retrospective	119	100**%**	37% > 60	50.4**%**	52.9**%**	CHOP-like 88.2% R	NR	NR
Kim et al. (2017) [35]	Retrospective	150	100**%**	mean 58.5 SD 14	57.3**%**	65.3**%**	R-CHOP21^a^	NR	NR
De Oliveira Costa et al. (2016) [36]	Prospective	111^b^/147	100**%**	58.9 (16–86)^c^	45.0**%**	64.0**%**	Stage I/II: 4× R- CHOP21^a^ + RT Stage III/IV: R-CHOP21^a^	43.2**%**	? refractory and relapsed pts.: IVAC +ASCT
Kong et al. (2016) [37]	Retrospective	105	100**%**	56 (19–82)	54.3**%**	43.8**%**	R-CHOP	NR	NR
Mikhaeel et al. (2016) [38]	Retrospective	147	100**%**	57 (22–86)	49.7**%**	68.7**%**	R-CHOP Stage I/II non-bulky: 3-4× R-CHOP and IFRT	34.0**%**	Not upfront (*n* = 1 after clinical progression)
Mamot et al. (2015) [39]	Prospective	125^b^/138	100**%**	58.4(18-81)^c^	54.3**%**^c^	53.6**%**^c^	R-CHOP14^d^ + 2R	17.4**%**^c^ =event	NR
Zhang et al. (2015) [40]	Retrospective	197	100**%**	46 (18–81)	60.4**%**	59.4**%**	14.2% R-CHOP14^d^ 85.8% R-CHOP21^a^	18.8**%**	Not upfront (*n* = 1 progression at end-of-treatment)
Carr et al. (2014) [41]	Prospective	327^b^/361	DLBCL: 97.2**%** PMBCL: 2.8**%**	55 (IQR 44–64)	52.9**%**	64.2**%**	(R-)CHOP21^a^ MACOP-B (*n* = 1) R-CNOP (*n* = 4) 86% R	20.2**%**	NR
Dabaja et al. (2014) [42]	Retrospective	294^b^/350	100**%**	49% > 61	55.4**%**	62.6**%**	82.0% R-CHOP 11.2% R-HCVAD 6.8% other	29.9**%**	NR
Mylam et al. (2014) [43]	Prospective	112	100**%**	62 (23–85)	52.7**%**	82.0**%**	84.8% R-CHOP 9.8% R-CHOEP 5.4% other (92.9% R 55% 14 day 45% 21 day)	In methods but no numbers	NR
Nols et al. (2014) [44]	Retrospective	73	100**%**	60 (18–85)	63.0**%**	68.5**%**	15.1% R-CHOP14^d^ 49.3% RCHOP21^a^ 11.0% R-mini-CHOP 23.3% R-ACVBP 1.4% CHOP	NR	8.2%
Fuertes et al. (2013) [45]	Prospective	50	100**%**	55 (21–79)	56.0**%**	44.0**%**	R-CHOP21^a^	NR	NR
Gonzalez-Barca et al. (2013) [46]	Prospective	69	100**%**	60 (18–78.9)	53.6**%**	65.2**%**	R-CHOP14^d^	5.8**%**	NR
Itti et al. (2013) [47]	Retrospective	114	100**%**	56 (23–80)	59.6**%**	82.5**%**	55.3% R-CHOP21^a^ 44.7% R-CHOP14^d^/ R-ACVBP	3.5**%**	? in young high- risk pts. as part of first-line consolidation or salvage
Lanic et al. (2012) [48]	Retrospective	45^b^/57	100**%**	65 (22–87)	48.9**%**	84.4**%**	75.5% R-CHOP 24.4% intensified R-CHOP	NR	8.9% frontline
Pregno et al. (2012) [49]	Retrospective	88	100**%**	55 (18–80)	46.6**%**	67.0**%**	35.2% R-CHOP21^a^ 64.8% R-CHOP14^d^	15.9**%**	NR
Safar et al. (2012) [50]	Retrospective	112	100**%**	59 (20–79)	67.0**%**	81.3**%**	50.9% R-CHOP21^a^ 21.4% R-CHOP14^d^ 27.7% R-ACVBP	0**%**	16.1% Consolidative HDT + ASCT (if <60 years + >1 aaIPI)
Cashen et al. (2011) [51]	Prospective	50	100**%**	Mean 58 (29–80)	NR	100**%**	R-CHOP21^a^	Upfront = Excl. crit. *n* = 1 after ASCT	Not upfront (*n* = 1 refractory disease and *n* = 2 progressive disease)
Zinzani et al. (2011) [52]	Retrospective	91	DLBCL: 85.7**%** PMBCL: 14.3**%**	54 (17–90)	52.7**%**	67.0**%**	DLBCL: 16.7% R-VNCOP-B (> = 60 yr) 83.3% R-CHOP21^a^ (<60 yr) PMBCL: 92.3% R-MACOP-B 7.7% R-CHOP21^a^	NR	Not upfront (7.7% with PR at end-of-treatment PET, 2.2% at time of relapse)
Zhao et al. (2007) [53]	Retrospective	32^e^ /61	DLBCL/PMBCL	57 (12^f^ −85)^c^	60.7**%** c	75.4**%** c	90.6% R-CHOP21^g^ 9.4% CHOP21^g^	Preplanned, depending on stage and site	NR

*Abbreviations: No* number, *RT* radiotherapy, *ASCT* autologous stem cell transplantation, *(R-)CHOP* (rituximab,) cyclophosphamide, doxorubicin, vincristine, prednisone, *R* rituximab, *NR* not reported, *SD* standard deviation, *IVAC* ifosfamide, mesna, cytarabine, etoposide, *IFRT* involved field radiotherapy, *PMBCL* primary mediastinal B-cell lymphoma, *IQR* interquartile range, *MACOP-B* methotrexate, cytarabine, cyclophosphamide,vincristine, prednisone, bleomycin, *CNOP* cyclophosphamide, mitoxantrone, vincristine, prednisone, *HCVAD* hyperfractionated cyclophosphamide, doxorubicin, vincristine, dexamethasone, *CHOEP* cyclophosphamide, doxorubicin, vincristine, etoposide, prednisone, *R-ACVBP* rituximab, doxorubicin, vindesine, bleomycin, prednisone, *pts.*  patients, *HDT* high dose therapy, *aaIPI* age adjusted international prognostic index, *Excl crit*  exclusion criterium, *VNCOP-B* etoposide, mitoxantrone, cyclophosphamide, vincristine, prednisone, bleomycin, *PR* partial response, *CVP* cyclophosphamide, vincristine, prednisone, *DHAP* dexamethasone, cytarabine, cisplatin, *MTX-AraC* methotrexate, cytarabine

^a^(R-)CHOP21: (rituximab,) cyclophosphamide, doxorubicin, vincristine, prednisone given with a 3 week interval between cycles

^b^Number of I-PET scans available for (*central*) review

^c^Only available for complete patient cohort

^d^(R-)CHOP14: (rituximab,) cyclophosphamide, doxorubicin, vincristine, prednisone given with a 2 week interval between cycles

^e^Data of DLBCL/PMBCL patients only (received from authors)

^f^Authors replied that only 1 patient with DLBCL was younger than 18 years old

^g^Authors replied that 29/32 DLBCL/PMBCL patients received R-CHOP21 and 3/32 received CHOP21

In Table 2 details of PET procedures, interpretation, and timing of interim PET between cycles are shown. Most studies performed an interim PET scan after two cycles of chemotherapy in all patients, one study made interim PET scans after only one course in all patients [43]; the remaining studies combined patient groups who had their interim assessment after a variable number of treatment cycles. The number of days after the previous treatment course at which the interim PET was acquired also varied between studies, mostly just before the next chemotherapy cycle, but the number of days after previous treatment was not reported by all studies. Twelve studies applied the Deauville scoring system and four the International Harmonization Project system [40, 46, 48, 51]. The remaining studies used a custom scoring system [42, 50, 52, 53].

**Table 2:** Interim ^18^F-FDG PET characteristics

First author, year	Timing		Interpretation		Scan Procedures				
	Interim ^18^F-FDG PET timing	Days after previous treatment median (range)	Criteria for positive scan	Interpreters	PET/CT or PET only	^18^F-FDG dose (MBq)	Uptake interval (min)	Fasting period (hours)	Glucose
Fan et al. (2017) [34]	2	NR	Deauville 4–5	3 NM	PET/CT	3.7/kg	60 + − 10	≥6	<200 mg/dL
Kim et al. (2017) [35]	2 or 3	19.7 + − 2.3	Deauville 4–5	2 NM	PET/CT	5.18/kg	50	≥6	NR
De Oliveira Costa et al. (2016) [36]	2	20	Deauville 4–5	1 NM + 1 RAD	Both	5/kg	60	≥6	<180 mg/dL
Kong et al. (2016) [37]	2	NR	Deauville 4–5	≥2 NM	PET/CT	5.5/kg	40–60	≥6	<=8 mmol/L
Mikhaeel et al. (2016) [38]	2	NR	Deauville 4–5	1 NM	PET/CT	370	90	6	NR
Mamot et al. (2015) [39]	2 (if positive also after 4	(11–14)	Deauville 4-5^a^	NR	Both	370	60	≥4	Measured before tracer injection
Zhang et al. (2015) [40]	2 (and 4)	NR	IHP	3 reviewers	PET/CT	4.4–7.4/kg	60–90	6	NR
Carr et al. (2014) [41]	2, 3 or 4	18 (IQR 17–21)	Deauville^b^	4 NM	Both	NR	NR	NR	NR
Dabaja et al. (2014) [42]	1, 2 or 3	Days from diagnosis 63 (21–126)	Custom^c^	2 physicians specializing in NM	PET/CT	average 630	mean 90 SD 22	≥6	<200 mg/dL
Mylam et al. (2014) [43]	1	Min 10 days	Deauville 4–5	2 out of 5 (random), discrep: 3rd	NR	NR	NR	NR	NR
Nols et al. (2014) [44]	3 or 4	NR	Deauville 4–5	2 NM from own dept	Both	200–365	97.5 + − 32.8	≥6	<175 mg/dL
Fuertes et al. (2013) [45]	2 or 3	18 (16–21)	Deauville 4–5	2 NM	PET/CT	296–444	60	4–6	<180 mg/dL
Gonzalez-Barca et al. (2013) [46]	2	2 days before 3rd cycle	IHP	NR	Both	3.7/kg	60–90	≥6	90–160 mg/dL
Itti et al. (2013) [47]	2	R-CHOP14/R-ACVBP: 13 + −2 R-CHOP 21: 19 + −4	Deauville 4–5	3 NM	PET/CT	5.4/kg 393 + −124	Median 69, mean 70 + − 16	fasting	<=11 mmol/L
Lanic et al. (2012) [48]	3 or 4	16 (3–27)	IHP	2 NM	PET/CT	5/kg	60	≥6	Range: 3.4–15.8 mmol/L
Pregno et al. (2012) [49]	2, 3 or 4	13 (4–27)	Deauville 4–5	2 NM	PET/CT	37 /10 kg	60 (*n* = 6 90 min)	≥6	90–160 mg/dL
Safar et al. (2012) [50]	2	Median 14	Custom^d^	1 NM	Both	2–5/kg	60 + − 10	≥6	Checked before the exam
Cashen et al. (2011) [51]	2 or 3	3 weeks	IHP	2 nuclear radiologists	PET/CT	370–555	+ − 60	fasting	<200 mg/dL (84–188)
Zinzani et al. (2011) [52]	3 or mid-treatment^e^	Immediately before subsequent cycle	Custom^f^	3 experienced readers	PET/CT	5.3/kg	60–90	6	NR
Zhao et al. (2007) [53]	3 or 4	Last day before new cycle	Custom^g^	2 NM	PET/CT	240–259	60	≥6	<7.8 mmol/L

*Abbreviations*: *MBq* megabecquerel, *min* minutes, *NR* not reported, *NM* nuclear medicine physician, *RAD* radiologist, *IHP* international harmonization project criteria, *IQR* interquartile range, *SD* standard deviation, *dept.* department, *(R-)CHOP* (rituximab,) cyclophosphamide, doxorubicin, vincristine, prednisone, *R* rituximab, *R-ACVBP* rituximab, doxorubicin, vindesine, bleomycin, prednisone

^a^Results presented for central review results only, local review results are based on SUVmax lesion > SUVmax of mediastinal blood pool

^b^Four categories; Negative/CR = resolution of abnormal ^18^F-FDG uptake at sites of disease identified on staging PET with any residual ^18^F-FDG uptake less than or equal to the mediastinal blood pool, CR-MRU = residual low-level ^18^F-FDG uptake at disease sites greater than mediastinum, but less than or equal to physiologic uptake in liver. Positive = residual or increased ^18^F-FDG uptake with intensity greater than liver at a site of known disease. Mixed response = reduction in ^18^F-FDG uptake at some disease sites, with increased ^18^F-FDG uptake at other existing or new sites

^c^Positive = according to a SUVmax measurement >2.5. Equivocal PET/CT or CT findings were then interpreted by using CT scan findings and clinical information to distinguish vascular/bowel activity from residual FDG uptake at the previously involved site

^d^A negative PET scan was defined as having no residual abnormal uptake or a minimal residual uptake. A positive scan was defined as having at least one residual site associated with an intensity markedly superior to local background as previously described (Haioun Blood 2005)

^e^After three cycles R-CHOP21 or midtreatment in case of R-VNCOP-B or R-MACOP-B

^f^Negative = no pathological tracer uptake was shown, unequivocally positive = areas of focal uptake localized to sites of previous disease (in this sense representing a residual disease or a disease relapse), within asymmetrical lymph nodes, or within lymph nodes unlikely to be affected by inflammation (mediastinal, except for hilar, and abdominal). Sites of known physiological uptake that showed symmetrical uptake were not described in the report

^g^Negative was defined as no evidence of disease. MRU was defined as low-grade uptake of FDG (just above background) in a focus within an area of previously noted disease reported by the nuclear medicine physicians. Positive was defined as increased uptake suspicious for malignant diseases, which did not have a benign explanation

The outcome measures of the included studies are shown in Table 3: 16 studies presented PFS and the other four studies reported EFS. The definitions of PFS and EFS for the different studies are presented in Supplemental Table 2. Percentages of positive interim PET scans ranged from 18.1 to 56.3%. Five original publications had reported univariate HRs, and four authors provided a (re)calculated HR upon our request. Two authors provided information about the number of events and *P*-values in order to use the method from Tierney et al. [28]. For one study we extracted the HR from the KM curves with numbers at risk provided by the authors and for six studies we used the KM curves without numbers at risk. For two studies we could not extract the HRs, as there was insufficient data and no Kaplan-Meier curve [36, 48].

**Table 3:** Study results; prognostic and diagnostic information

First author, year	Results			Prognostic information		Diagnostic information at 2 years^a^			
	Outcome measure	Median follow-up in months (range)	Interim PET positive no (%)	HR uni- variate	95% CI	Sensitivity (95% CI)	Specificity (95% CI)	Positive predictive value (95% CI)	Negative predictive value (95% CI)
Fan et al. (2017) [34]	PFS	26 (2–75)	42 (35.3%)	4.46^b^	2.42-8.23^b^	60% (46–74)	79% (69–87)	62% (47–75)	78% (67–86)
Kim et al. (2017) [35]	PFS	31 (8–75)	43 (28.7%)	1.81^b^	0.95-3.45^b^	38% (25–54)	75% (66–82)	35% (22–50)	78% (69–84)
De Oliveira Costa et al. (2016) [36]	PFS	41.5 (0.6–71.1)	51 (45.9%)	NR	NR	59% (36–78)	56% (46–66)	20% (11–32)	88% (78–94)
Kong et al. (2016) [37]	PFS	32 (9–59)	19 (18.1%)	9.74^c^	3.27–28.99^c^	64% (43–80)	94% (87–97)	74% (51–88)	91% (83–95)
Mikhaeel et al. (2016) [38]	PFS	45.6 (15.6–94.8)	65 (44.2%)	2.18^c^	1.11–4.27^c^	63% (48–76)	63% (54–72)	42% (30–54)	80% (71–88)
Mamot et al. (2015) [39]	EFS	45 (4–64)^d^	58 (46.4%)	3.23^d^	1.78–5.89^d^	68% (54–79)	68% (57–77)	59% (46–70)	76% (65–85)
Zhang et al. (2015) [40]	PFS	30 (5–94)	87 (44.2%)	2.74^c^	1.52–4.93^c^	70% (59–79)	72% (63–79)	60% (49–69)	80% (72–86)
Carr et al. (2014) [41]	EFS	35 (75% at least 24 mo)	117(35.8%)	5.31^b^	3.29–8.56^b^	70% (58–79)	74% (68–79)	42% (33–51)	90% (85–93)
Dabaja et al. (2014) [42]	PFS	36 (0.8–84)	54 (18.4%)	1.9^b^	1.1–3.2^b^	33% (20–46)	85% (80–89)	33% (22–47)	85% (79–89)
Mylam et al. (2014) [43]	PFS	29 (2–80)	60 (53.6%)	1.45^e^	0.71–2.97^e^	64% (43–80)	49% (39–59)	23% (14–35)	85% (72–92)
Nols et al. (2014) [44]	PFS	29 (3.3–86)	20 (27.4%)	5.26^f^	1.90–14.58^f^	58% (36–77)	83% (71–91)	55% (34–74)	85% (73–92)
Fuertes et al. (2013) [45]	PFS	Surviving: 46.8 (2.4–78)	12 (24.0%)	3.89^c^	1.07–14.22^c^	46% (23–71)	84% (69–92)	50% (25–75)	82% (67–91)
Gonzalez-Barca et al. (2013) [46]	EFS	28.8 (5.8–52.6)	34 (49.3%)	2.70^c^	0.68–10.67^c^	75% (46–91)	56% (43–68)	26% (15–43)	91% (78–97)
Itti et al. (2013) [47]	PFS	Living: 39 (12–74) Relapse: 6 (1–32) Death: 17 (3–55)	51 (44.7%)	2.85^d^	1.38–5.87^d^	68% (49–82)	63% (52–72)	37% (25–51)	86% (75–92)
Lanic et al. (2012) [48]	PFS	27 (7–73)	14 (31.1%)	NR	NR	NR	NR	NR	NR
Pregno et al. (2012) [49]	PFS	26.2 (8–67)	25 (28.4%)	2.45^b^	1.01–5.93^b^	44% (23–67)	75% (64–84)	28% (14–47)	86% (75–92)
Safar et al. (2012) [50]	PFS	Living: 38 (4.4–73)	42 (37.5%)	4.77^d^	2.26–10.05^d^	72% (54–85)	75% (64–83)	50% (35–64)	89% (79–94)
Cashen et al. (2011) [51]	PFS	Surviving: 33.9 (16–44)	24 (48.0%)	2.98^d^	1.16–7.67^d^	69% (42–87)	59% (43–74)	38% (21–57)	85% (66–94)
Zinzani et al. (2011) [52]	EFS	No events: 50 (12–68)	35 (38.5%)	3.94^c^	1.69–9.19^c^	87% (68–95)	78% (67–86)	57% (41–72)	95% (85–98)
Zhao et al. (2007) [53]	PFS	27 (9–45)^g^	18 (56.3%)	3.67^e^	1.61–8.35^e^	72% (49–88)	64% (39–84)	72% (49–88)	64% (39–84)

*Abbreviations*: *95% CI* 95% confidence interval, *PFS* Progression-free survival, *NR* Not reported, *EFS* Event-free survival, *mo* months

^a^Extracted predictive test accuracy measures at 2 years survival prediction in Kaplan-Meier curve

^b^Reported in publication

^c^Extracted from KM without numbers at risk

^d^Reply on request for additional information

^e^Tierney method based on number of events and *P*-value

^f^Extracted from KM with numbers at risk

^g^Only available for complete patient cohort

**Fig. 2:**
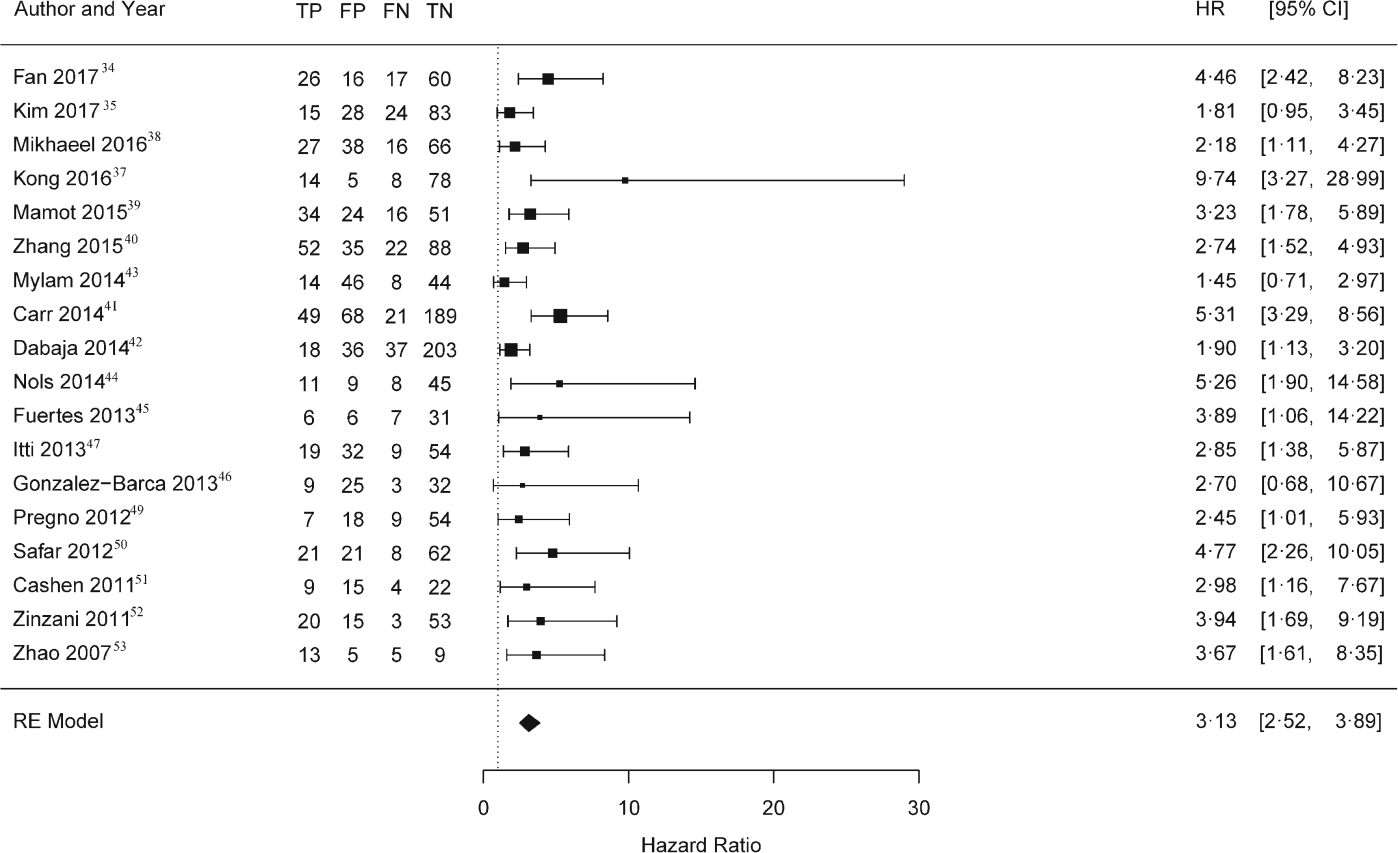
Forest plot of univariate hazard ratios for interim PET scans in diffuse large B-cell lymphoma. This plot shows the univariate hazard ratios (black squares, size based on study size), and 95% CI’s (horizontal lines) of the individual studies sorted by publication year for PFS/EFS of the interim PET positive and negative patients. The estimated pooled effect estimation is shown with a diamond. For each study a 2 × 2 contingency table at 2 years follow-up is shown

In Fig. 2 the Forest plot with the 18 univariate HRs is shown. The pooled effect estimate was 3.13 (95% CI 2.52–3.89). The Cochran’s Q test for heterogeneity was not statistically significant (*P* = 0.087) and between study heterogeneity was low (I^2^ = 35.14%). The 95% prediction interval was 1.68–5.83, with one outlier [37].

The methodological quality was assessed based on the QUADAS-2 and QUIPS checklists. Subgroup analyses were performed on study design characteristics that were potential sources of bias.

Meta-regression showed that the outcomes did not differ between retrospective and prospective studies, studies with blinded review and studies that did not report whether they blinded the PET/CT assessment, or studies that used PFS or EFS as outcome measure. A statistically significant higher HR was found for studies with a combination of integrated PET/CT- and PET standalone systems compared to studies with integrated PET/CT systems only (HR 4.39 vs 2.85*, P* = 0.0332) and a trend towards a higher HR in studies with 80–99% DLBCL compared to studies with 100% DLBCL (*P* = 0.0577). Prespecified subgroups for different types of treatments and FDG-PET scoring systems showed no statistically significant differences (Supplemental Table 3). For the subgroups “availability of baseline PET or CT” and “central or local review procedure”, insufficient information was reported to perform these analyses. Risk of publication bias as assessed with a Funnel plot was low (Supplemental Fig. 1).

Nineteen studies had data available for the calculation of PPV, NPV, sensitivity, and specificity of interim PET for prediction of two-year-PFS or -EFS. For one study we could not extract or calculate the diagnostic measures [48]. PPV and NPV ranged from 20 to 74% and 64 to 95%, respectively. Sensitivity and specificity ranged from 33 to 87% and 49 to 94%, respectively (Table 3**,** Supplemental Fig. 2).

**Fig. 3:**
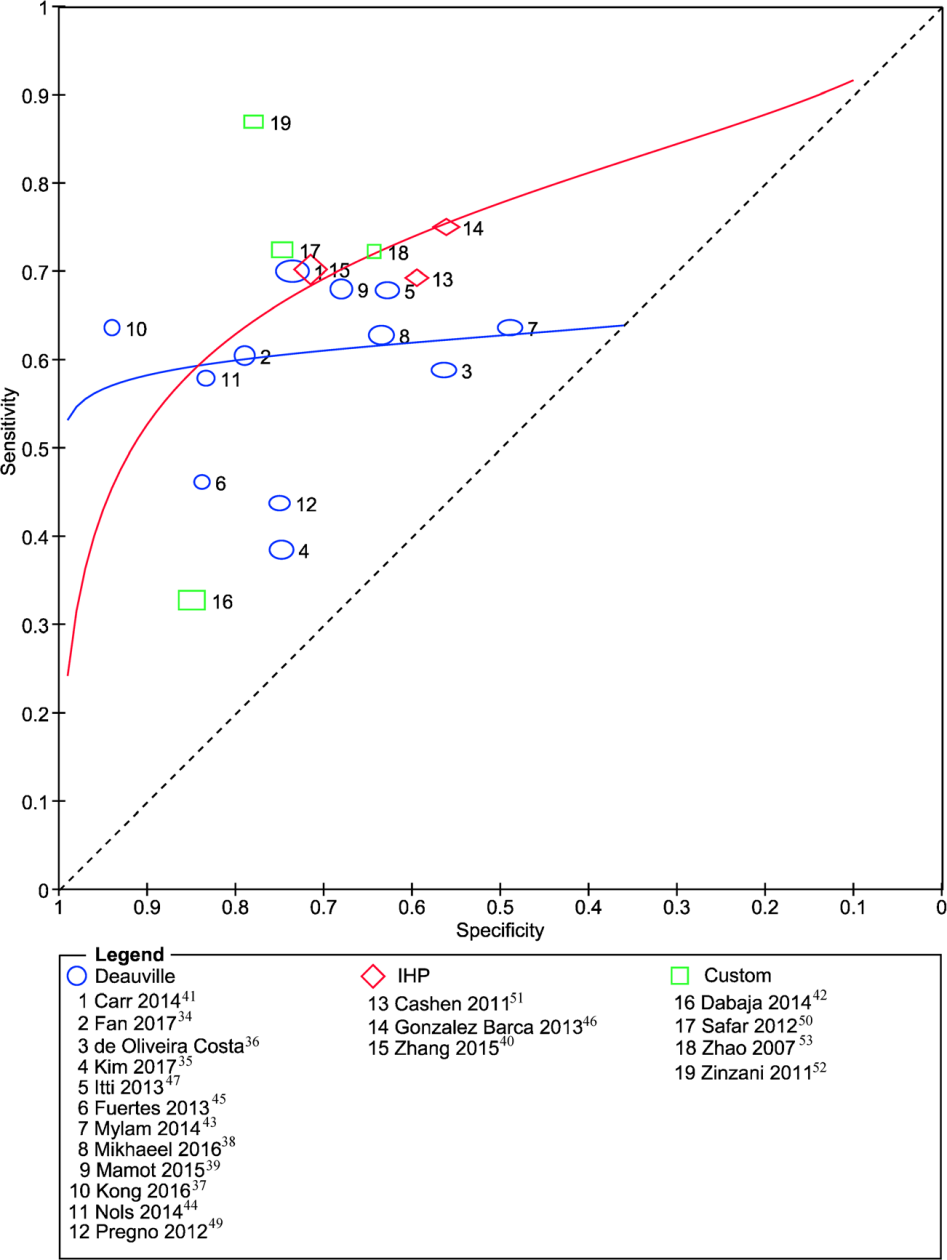
Summary receiver operating curves (sROC) for different visual interim PET criteria. Studies that assessed interim PET scans according to the Deauville’s criteria are indicated with *blue circles*, studies that used the international harmonization project (IHP) criteria are indicated with *red diamonds* and studies that used custom visual criteria are indicated with *green squares*. The size of the *circles*, *diamonds*, and *squares* are based on the inverse standard error

In Fig. 3 the ROC curves of the different visual criteria are shown. The studies that were classified as “custom”, did not have comparable scan positivity definitions and therefore no summary curve for this group was presented. We found no statistically significant differences between the curves for Deauville and IHP. There was a trend (*P* = 0.0503) towards a higher accuracy for studies with DLBCL 80–99% versus studies with 100% DLBCL patients.

## Discussion

This systematic review and meta-analysis included 20 studies comprising a total of 2,411 DLBCL patients who underwent interim ^18^F-FDG PET. Eighteen studies were eligible for the HR and 19 for the HSROC meta-analyses. We found a pooled estimated HR of 3.13 (95% CI 2.52–3.89) for interim PET in the prediction of PFS or EFS. The prediction interval ranged from 1.68 to 5.83, suggesting that a new study investigating the prognostic value of interim PET on PFS or EFS will find a HR in this range with 95% confidence. These results confirm the predictive value of interim PET in DLBCL patients for PFS and EFS. Our pooled estimated HR was lower than reported in a previous meta-analysis (2013) [16] which reported a pooled estimated HR of 4.4 (95% CI 3.34–5.81) from nine studies investigating the prediction of PFS by interim PET. They used a similar approach to extract HRs; however, they had less strict inclusion criteria with regard to the NHL types and follow-up period, both visual and semi-quantitatively assessed PET scans were included, and no subgroup analyses were performed. Despite these differences, their HR result is within the range of our calculated 95% prediction interval and the amount of statistical heterogeneity (I^2^ = 39%) amongst studies was comparable. Other meta-analyses did not compare the HRs between studies [15, 17, 18].

We have no explanation for the statistically significant higher HR for studies (*n* = 5) that used both PET/CT- and PET standalone systems compared to studies that used an integrated PET/CT system.

The trend towards a higher HR for the studies with both DLBCL and PMBCL patients compared to studies with only DLBCL patients could not directly be explained by the inclusion of both lymphoma subtypes. The fact that two out of three studies with both DLBCL and PMBCL patients [52, 53] used custom criteria for the interpretation of the interim PET could possibly explain this. These meta-regression results should be interpreted with caution, as the number of studies per subgroup were relatively low (Supplemental Table 3) which precludes multivariate meta-regression analysis.

Diagnostic 2 × 2 contingency tables of interim PET showed wide ranges between studies for sensitivity, specificity, and positive predictive values at 2 years. The ranges reported in other systematic reviews and meta-analyses were hard to compare as they used the complete follow-up period for their calculations, included studies with follow-up periods less than 24 months, and used other statistical methods [15, 17, 18]. We decided to truncate at 2 years, as most clinically relevant events occur during this period. Moreover, the widely ranging complete follow-up periods of individual studies might introduce bias.

Negative predictive values for 2-year progression-free status were generally above 80%, except in four studies [34, 35, 39, 53]. In Mamot et al. [39], the somewhat lower negative predictive value could possibly be explained because radiotherapy (administered regardless of PET results) was counted as an event and resulted in a lower EFS rate compared to other clinical trials. Zhao et al. [53] had a low percentage of negative interim PET scans and a high number of events, which explains the lower NPV.

The higher sensitivity values seen in ROC analysis for both IHP and custom criteria vs. the Deauville system may be explained by the lower threshold of test positivity with IHP vs. Deauville (using liver and blood pool activity as the reference tissue, respectively). None of the studies using custom criteria defined a threshold comparable to or higher than hepatic uptake. We found widely ranging positivity rates between studies, which are mainly in agreement with the timing of interim PET between cycles and the criteria used. In an exploratory analysis on five studies [34, 37–39, 47] that performed interim PET strictly after 2 cycles of therapy and applied the Deauville scoring system we found a pooled estimated HR of 3.48 (95% CI 2.46–4.93) with a corresponding 95% prediction interval of 1.58–7.67 (Supplemental Fig. 3). The positivity rates for these studies ranged between 18 and 46%, PPV from 37 to 74% and NPV from 76 to 91%, comparable to the analysis including all studies.

We chose to present the methodological characteristics along the other characteristics of the study population and treatments (Table 1) and along characteristics (including timing between cycles) of the index test (Table 2).

QUADAS-2 and QUIPS criteria were applied to assess the quality of the studies from the perspective of risk of bias and applicability. In this review, the strict inclusion and exclusion criteria with regard to patient population (>80% DLBCL), index test (interim PET between one and five treatment cycles), and reference standard (PFS and EFS) guaranteed the applicability of the results to the review question. In the subgroup analyses we examined whether bias could have occurred because of methodological shortcomings. It appeared that none of these affected the results. Only characteristics of the population (< 100% DLBCL) and a combination of integrated and standalone systems seemed to have impact on the predictive value of interim PET.

We used a comprehensive search strategy and applied strict inclusion and exclusion criteria. We focused on DLBCL patients, and 2-year PFS. Moreover, we examined the influence of different design characteristics (retrospective and prospective, blinded review or not reported; PFS or EFS), characteristics of patients (100% DLBCL or between 80 and 100%), treatments (ASCT upfront or not, preplanned or consolidative radiotherapy used or unknown), availability of a baseline PET or CT, properties of scans (PET/CT or a combination of PET/CT and PET standalone systems), and scoring issues (DS -, IHP -, or custom criteria, central review or local review). Only the patient characteristics and properties of scans affected the results. It appeared that the HR estimates of the included studies were quite homogeneous (I^2^ = 35%).

By contacting the authors we were able to include most of the eligible studies in our meta-analysis and deducting data that was not presented by the authors directly. Some data though were hard to obtain from the studies.

First of all, the definition of the start of the progression-free survival and event-free survival differed amongst studies. Some studies started their follow-up period at the time from diagnosis and others from initiation of first-line treatment. Recently some data has shown that patients who have a more aggressive disease tend to be treated earlier, so there could be selection bias between studies that have a shorter period between time of diagnosis and initiation of treatment versus studies with a longer period [54]. For future studies it seems important to have a comparable start of the follow-up period and authors should report the interval between diagnosis and start of the treatment to prevent or adjust for this risk of bias.

Another issue is that timing of the interim PET scans between cycles was different between studies; not only did the timing after which cycle the scan is performed differ, but also the number of days between the previous treatment course and interim PET. Unfortunately, not all authors report on this, although it is recommended to perform the scan at least 10 days after the previous course of chemotherapy, because of possible effects on tumor metabolism and systemic effects by, for example, growth factors [55].

In systematic reviews, investigators need to make choices. We chose to use the univariate data. This choice was made because univariate data were available in most studies and because of the large heterogeneity in factors for which the HR was adjusted in the primary articles. The adjusted factors were limited by the low number of events in most studies and partially based on available information such as quantitative PET analyses, immunohistochemistry and collection of specific clinical data (e.g. bone marrow involvement). Fourteen of the 20 studies performed a multivariate analysis. Most articles adjusted for the IPI score [34–39, 41–43, 46] or age-adjusted IPI [44, 48, 49], some dichotomized the score and others used the individual components. Results were varying widely; in some studies both interim PET and (aa)IPI showed an independent association with PFS or EFS [42, 48], others only for interim PET [34, 37, 39, 41, 44, 53], or (aa)IPI [43, 49] or no independent associations were found for both interim PET or (aa)IPI [35, 36, 38, 46]. One could argue that reporting univariate HRs instead of multivariate HRs could result in an overestimation of the predictive value of interim PET. Three studies reported both uni- and multivariate HRs and differences between univariate and multivariate HRs were −0.99 [41], 0.0 [39],and + 0.2 [42], respectively.

We further decided to choose the DS threshold for the interim response criteria which is most commonly described (DS < = 3 versus DS > = 4), because presenting all thresholds would increase heterogeneity, influence effect sizes, and finally use the same patients data multiple times in the analyses. Four studies presented multiple scores. Mylam et al. [43] published data about positivity for Deauville scores 4 and 5 as well as for Deauville score 5 and for IHP. Kim et al. [35] and Itti et al. [47] presented data about different positivity cutoff values for Deauville scores. Fuertes et al. [45] published a regular Deauville score as well as a 3 point-scale. In this review, we focused on visual response assessment criteria, and the potential added value of quantitative PET metrics is currently being investigated. Recently, a large phase III PET-adapted trial showed in a post-hoc analysis that a SUVmax reduction strategy [56] seems to discriminate better between good and poor outcome compared to the Deauville scoring system [57]. Finally, it should be mentioned that the studies from Safar et al. [50] and Itti et al. [47] had a small overlap in patient inclusion (*n* = 7); however, this will presumably not bias our results due to the small number.

## Conclusion

This systematic review and meta-analysis shows that interim PET in DLBCL patients has predictive value (HR 3.13). However, some diagnostic test characteristics are still too low, especially the positive predictive value should be improved, before a risk stratified treatment approach can be implemented in clinical practice.

## Electronic supplementary material

ESM 1 (PNG 137 kb)

High resolution image (TIFF 102029 kb)

ESM 2 (PNG 167 kb)

High resolution image (TIF 93.5 mb)

ESM 3 (PNG 119 kb)

High resolution image (TIFF 2.76 mb)

ESM 4 (DOCX 226 kb)
