# Supplementary figures and images for: Predictive value of interim positron emission tomography in diffuse large B-cell lymphoma: a systematic review and meta-analysis
Source: Eur J Nucl Med Mol Imaging. 2018 Aug 23;46(1):65–79. doi: 10.1007/s00259-018-4103-3 (PMC6267696; doi:10.1007/s00259-018-4103-3)

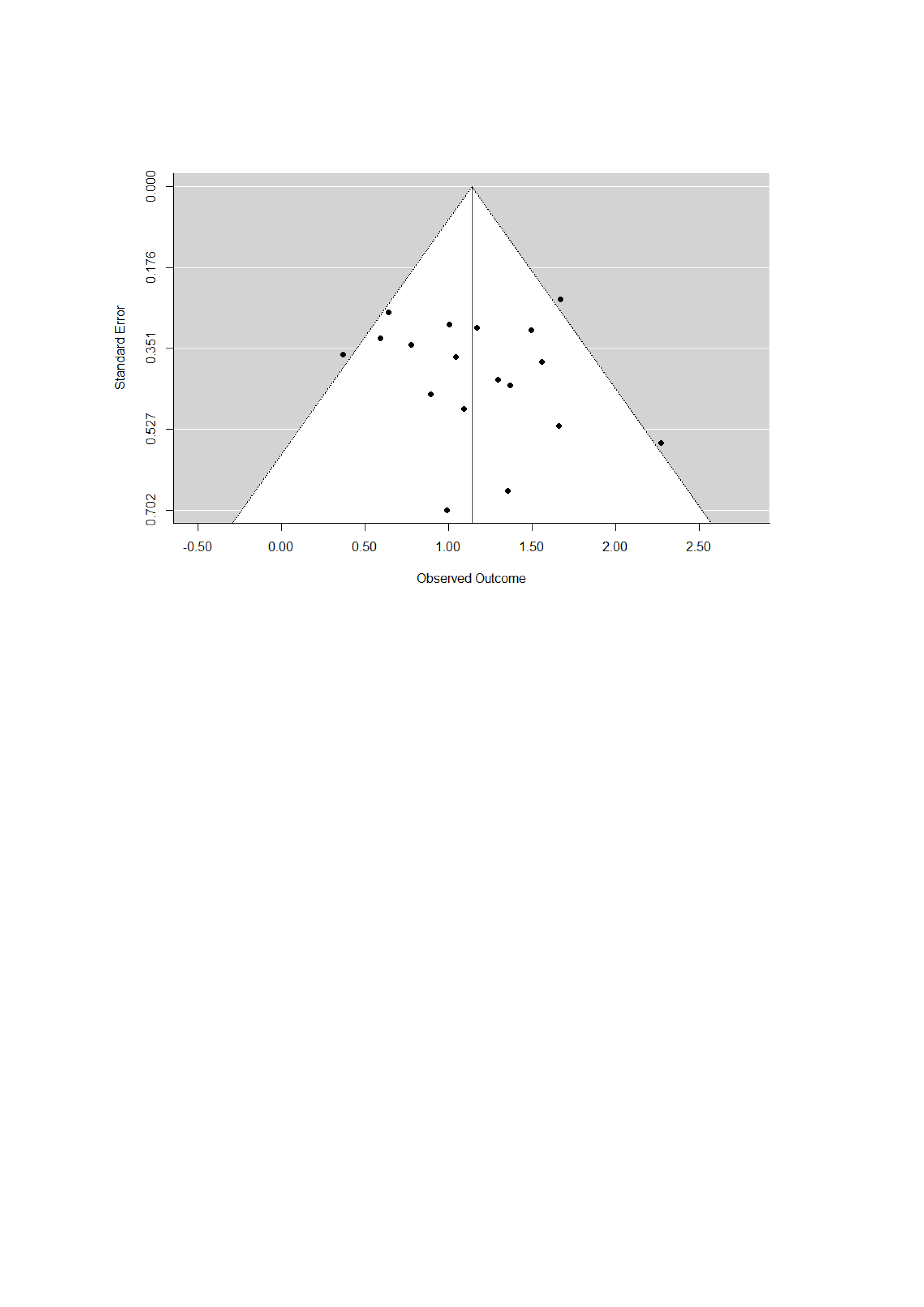

Supplement: Supplementary file 1 — (PNG 137 kb) [file 259_2018_4103_Fig4_ESM.png]

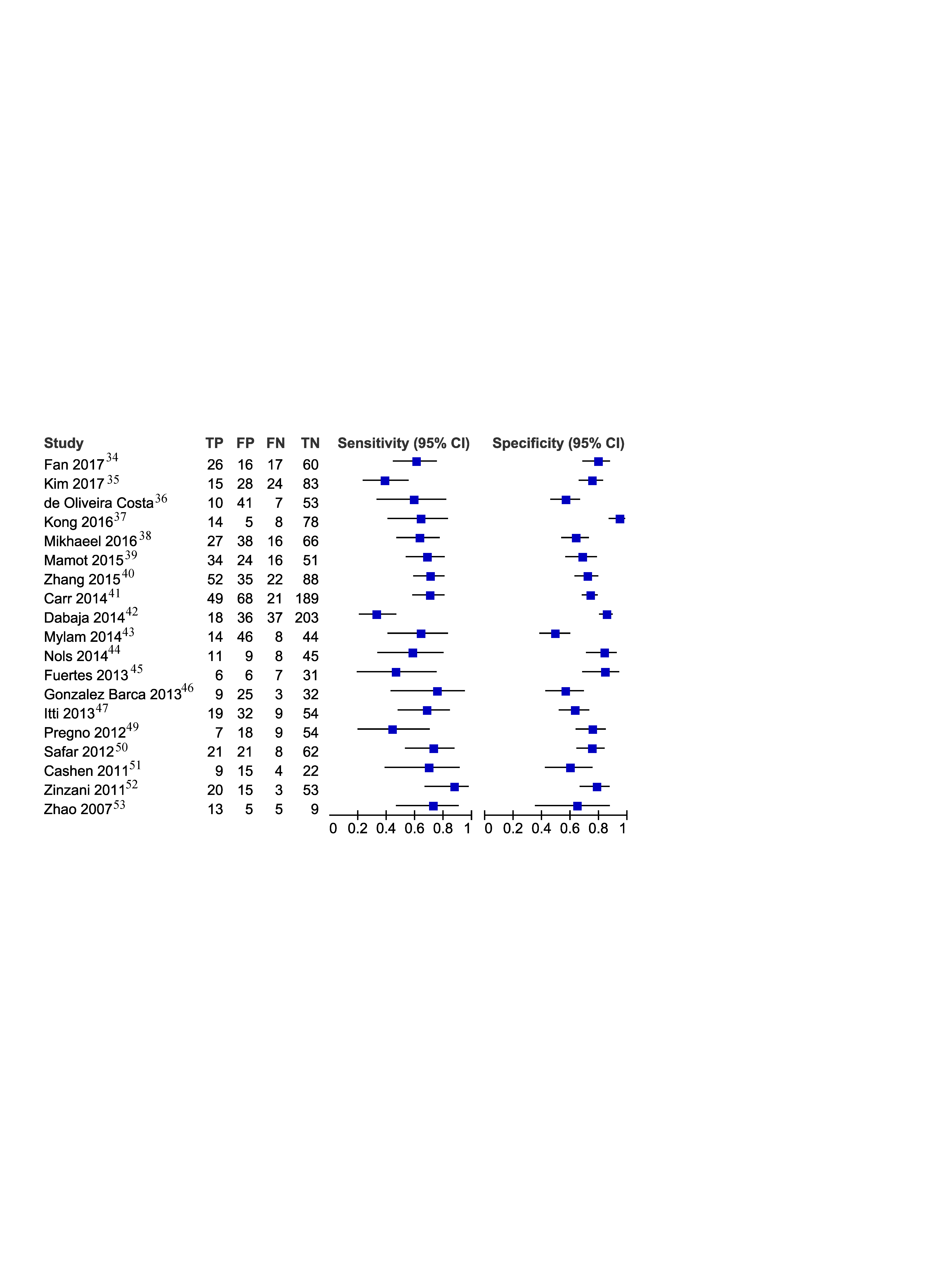

Supplement: Supplementary file 3 — (PNG 167 kb) [file 259_2018_4103_Fig5_ESM.png]

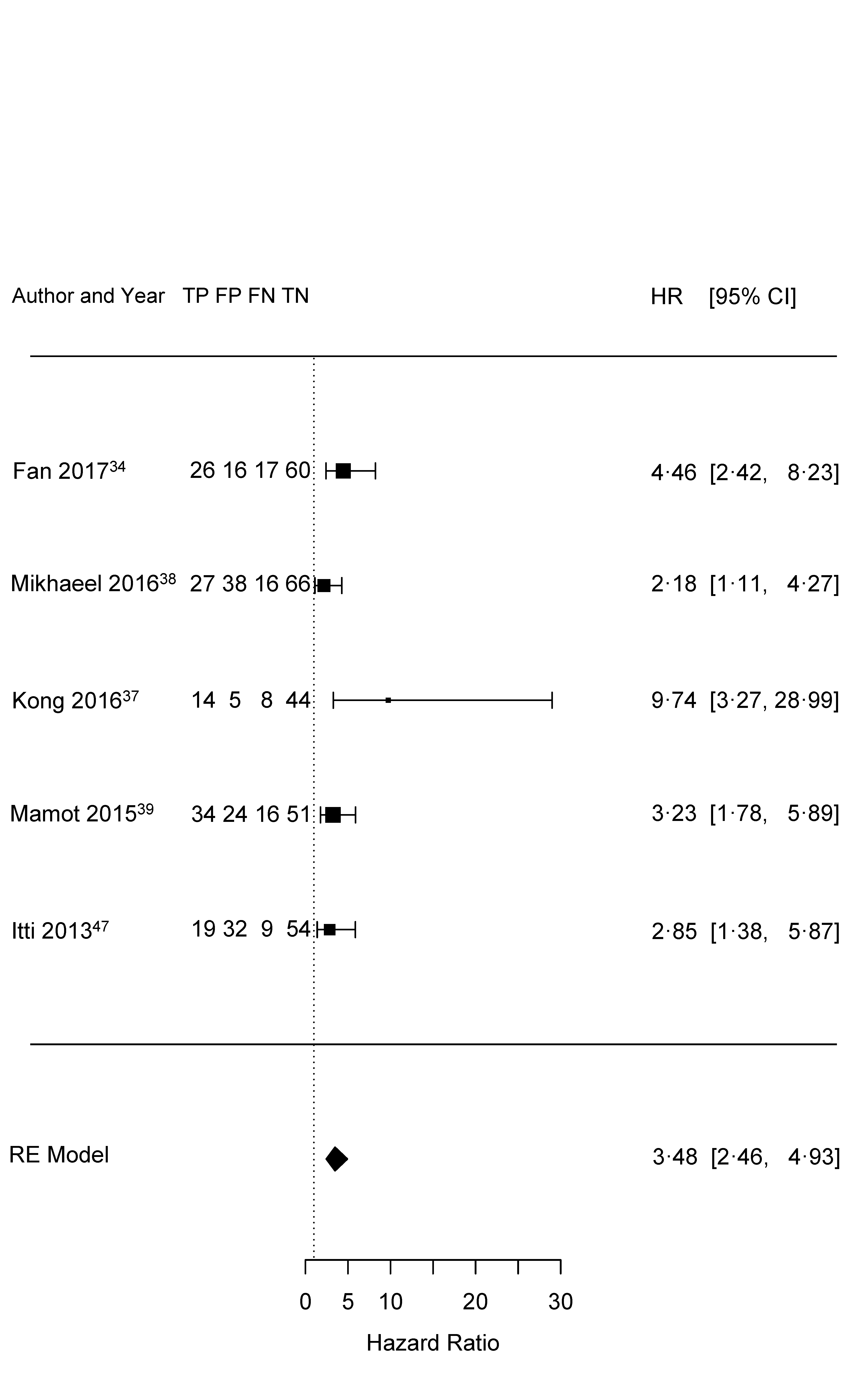

Supplement: Supplementary file 5 — (PNG 119 kb) [file 259_2018_4103_Fig6_ESM.png]
