# Supplementary material for: Predictive value of interim positron emission tomography in diffuse large B-cell lymphoma: a systematic review and meta-analysis
Source: Eur J Nucl Med Mol Imaging. 2018 Aug 23;46(1):65–79. doi: 10.1007/s00259-018-4103-3 (PMC6267696; doi:10.1007/s00259-018-4103-3)
Supplement: Supplementary file 7 — (DOCX 226 kb) [file 259_2018_4103_MOESM4_ESM.docx]

**Supplemental materials**

**Supplemental Table 1**

A. Pubmed/MEDLINE search strategy

| **Search** | **Query** | **Items found** |
| --- | --- | --- |
| #1 | "Lymphoma, Non-Hodgkin"[Mesh] OR Lymphoma*[tiab] OR Non-Hodgkin*[tiab] OR Non Hodgkin*[tiab] OR nonhodgkin*[tiab] OR NHL[tiab] OR DLBCL[tiab] OR Lymphoma*[ot] OR Non-Hodgkin*[ot] OR Non Hodgkin*[ot] OR nonhodgkin*[ot] OR NHL[ot] OR DLBCL[ot] | 182131 |
| #2 | ("Tomography, Emission-Computed"[Mesh:NoExp] OR "Positron-Emission Tomography"[Mesh] OR deoxyglucose[MeSH] OR deoxyglucose[tiab] OR desoxyglucose[tiab] OR deoxy-glucose[tiab] OR desoxy-glucose[tiab] OR deoxy-d-glucose[tiab] OR desoxy-d-glucose[tiab] OR 2deoxyglucose[tiab] OR 2deoxy-d-glucose[tiab] OR fluorodeoxyglucose[tiab] OR fluorodesoxyglucose[tiab] OR fludeoxyglucose[tiab] OR fluordeoxyglucose[tiab] OR fluordesoxyglucose[tiab] OR 18fluorodeoxyglucose[tiab] OR 18fluorodesoxyglucose[tiab] OR Fluoro-d-glucose[tiab] OR Fludeoxyglucose[tiab] OR Fluordeoxyglucose[tiab] OR 18fluordeoxyglucose[tiab] OR fdg*[tiab] OR 18fdg*[tiab] OR 18f-dg*[tiab] OR 18f-fdg[tiab] OR SUV[tiab] OR ((fluor[tiab] OR 2fluor*[tiab] OR fluoro[tiab] OR fluorodeoxy[tiab] OR fludeoxy[tiab] OR fluorine[tiab] OR 18f[tiab] OR 18flu*[tiab] AND glucose[tiab])) AND (pet[tiab] OR pet/*[tiab] OR petscan*[tiab] OR "Tomography, Emission-Computed"[Mesh:NoExp] OR "Positron-Emission Tomography"[Mesh] OR (emission[tiab] AND tomograph[tiab] OR tomographs[tiab] OR tomographic*[tiab] OR tomography[tiab] OR tomographies[tiab] OR scan[tiab]))) OR (deoxyglucose[ot] OR desoxyglucose[ot] OR deoxy-glucose[ot] OR desoxy-glucose[ot] OR deoxy-d-glucose[ot] OR desoxy-d-glucose[ot] OR 2deoxyglucose[ot] OR 2deoxy-d-glucose[ot] OR fluorodeoxyglucose[ot] OR fluorodesoxyglucose[ot] OR fludeoxyglucose[ot] OR fluordeoxyglucose[ot] OR fluordesoxyglucose[ot] OR 18fluorodeoxyglucose[ot] OR 18fluorodesoxyglucose[ot] OR Fluoro-d-glucose[ot] OR Fludeoxyglucose[ot] OR Fluordeoxyglucose[ot] OR 18fluordeoxyglucose[ot] OR fdg*[ot] OR 18fdg*[ot] OR 18f-dg*[ot] OR 18f-fdg[ot] OR SUV[ot] OR ((fluor[ot] OR 2fluor*[ot] OR fluoro[ot] OR fluorodeoxy[ot] OR fludeoxy[ot] OR fluorine[ot] OR 18f[ot] OR 18flu*[ot] AND glucose[ot])) AND (pet[ot] OR pet/*[ot] OR petscan*[ot] OR "Tomography, Emission-Computed"[Mesh:NoExp] OR "Positron-Emission Tomography"[Mesh OR (emission[ot] AND tomograph[ot] OR tomographs[ot] OR tomographic*[ot] OR tomography[ot] OR tomographies[ot] OR scan[ot]))) | 77819 |
| #3 | #1 AND #2 | 4042 |

B. Embase search strategy

| **Search** | **Query** | **Items found** |
| --- | --- | --- |
| #1 | 'nonhodgkin lymphoma'/exp OR lymphoma*:ab,ti OR (non NEXT/1 hodgkin*):ab,ti OR nonhodgkin*:ab,ti OR nhl:ab,ti OR dlbcl:ab,ti | 258953 |
| #2 | 'emission tomography'/de OR 'positron emission tomography'/exp OR 'whole body pet'/exp OR 'deoxyglucose'/exp OR deoxyglucose:ab,ti ORdesoxyglucose:ab,ti OR 'deoxy-glucose':ab,ti OR 'desoxy glucose':ab,ti OR 'deoxy-d-glucose':ab,ti OR 'desoxy-d-glucose':ab,ti OR2deoxyglucose:ab,ti OR '2deoxy-d-glucose':ab,ti OR fluorodeoxyglucose:ab,ti OR fluorodesoxyglucose:ab,ti OR fluordesoxyglucose:ab,ti OR 18fluorodeoxyglucose:ab,ti OR 18fluorodesoxyglucose:ab,ti OR 'fluoro d glucose':ab,ti OR fludeoxyglucose:ab,ti OR fluordeoxyglucose:ab,ti OR 18fluordeoxyglucose:ab,ti OR fdg*:ab,ti OR 18fdg*:ab,ti OR '18f-dg':ab,ti OR '18f fdg':ab,ti OR suv:ab,ti OR (fluor:ab,ti OR 2fluor*:ab,ti ORfluoro:ab,ti OR fluorodeoxy:ab,ti OR fludeoxy:ab,ti OR fluorine:ab,ti OR 18f:ab,ti OR 18flu*:ab,ti AND glucose:ab,ti) AND (pet*:ab,ti OR 'emission tomography'/de OR 'positron emission tomography'/exp OR 'whole body pet'/exp OR (emission:ab,ti AND (tomograph:ab,ti OR tomographs:ab,ti OR tomographic*:ab,ti OR tomography:ab,ti OR tomographies:ab,ti OR scan:ab,ti))) | 136600 |
| #3 | #1 AND #2 | 9470 |
| #4 | #3 AND ('article'/it OR 'article in press'/it OR 'review'/it) | 5648 |

C. Cochrane Library search strategy

| **Search** | **Query** | **Items found** |
| --- | --- | --- |
| #1 | Lymphoma* or "Non-Hodgkin*" or "Non Hodgkin*" or nonhodgkin* or NHL or DLBCL:ti,ab,kw (Word variations have been searched) | 7736 |
| #2 | Tomography or deoxyglucose or desoxyglucose or deoxy-glucose or desoxy-glucose or deoxy-d-glucose or desoxy-d-glucose or 2deoxyglucose or 2deoxy-d-glucose or fluorodeoxyglucose or fluorodesoxyglucose or fludeoxyglucose or fluordeoxyglucose or fluordesoxyglucose or 18fluorodeoxyglucose or 18fluorodesoxyglucose or Fluoro-d-glucose or Fludeoxyglucose or Fluordeoxyglucose or 18fluordeoxyglucose or fdg* or 18fdg* or 18f-dg* or 18f-fdg or SUV or PET*:ti,ab,kw (Word variations have been searched) | 26052 |
| #3 | #1 AND #2 | 502 |

**Supplemental Table 2** PFS/EFS definitions

| **First author (year)** | **PFS/EFS; definition** |
| --- | --- |
| Fan et al (2017)^34^ | PFS; was calculated *from the date of acquisition of biopsy results* to disease progression, relapse, death of patients from any causes, or the date of last follow-up for surviving ones. |
| Kim et al (2017)^35^ | PFS; was calculated *from the start of the treatment* to disease progression, recurrence or death. |
| De Oliveira Costa et al (2016)^36^ | PFS; was defined as the *time from the date of diagnosis* to the date of disease progression, relapse, or death as a result of any cause or last patient follow-up. |
| Kong et al (2016)^37^ | PFS; was defined as the interval between the *date of diagnosis* and the date of lymphoma progression, first relapse, death from any cause, or the last follow-up date. |
| Mikhaeel et al (2016)^38^ | PFS; defined as the *time from diagnosis* to the point of progression or death from any cause. Patients still alive were censored at the date of last contact. |
| Mamot et al (2015)^39^ | EFS; The primary end point was 2-year EFS *from start of treatment*. Patients who progressed, relapsed, switched to other treatments (including concomitant radiotherapy), refused to continue trial treatment, or died within 2 years were considered to have treatment failure. |
| Zhang et al (2015)^40^ | PFS; was defined as the *time from the start of treatment* to the progression of lymphoma, death from any cause, or last follow-up. |
| Carr et al (2014)^41^ | EFS; Study events were relapse after complete remission; death from any cause; treatment escalation for progressive disease while on treatment, and disease progression or failure to achieve complete remission at end-chemotherapy based on the revised response criteria for PET, with confirmation by biopsy that residual or increased ^18^F-FDG uptake was due to lymphoma. Cases lost to follow-up were censored at date of last known disease status. *Date of first treatment as origin*. |
| Dabaja et al (2014)^42^ | PFS; was defined as the *time from diagnosis* until objective tumor progression or death. |
| Mylam et al (2014)^43^ | PFS; was defined as the *time from diagnosis* to DLBCL progression or death from any cause. Patients who were still alive at the end of the study were censored at the date of data collection. |
| Nols et al (2014)^44^ | PFS; was defined as the *time from study entry* until disease progression or death due to any cause. |
| Fuertes et al (2013)^45^ | PFS; was defined as the interval *from the start of treatment* until disease progression of DLBCL, death from any cause or the most recent follow-up. |
| Gonzalez-Barca et al (2013)^46^ | EFS; An event was defined as follows: nonachievement of a CR or Cru with treatment, relapse after achievement of complete remission, or death from any cause, whichever came first. |
| Itti et al (2013)^47^ | PFS; calculated *from the date of diagnosis* until relapse with censoring at the time of last follow-up. |
| Lanic et al (2012)^48^ | PFS; was calculated *from the date of enrollment* until disease progression, relapse or death (from any cause) or last patient follow-up. |
| Pregno et al (2012)^49^ | PFS; was defined as the time *from the start of treatment* to death/progression as a result of any cause; patients still alive were censored at the date of last contact. |
| Safar et al (2012)^50^ | PFS; was calculated *from the date of enrollment* until disease relapse, with censoring at the time of last follow-up. |
| Cashen et al (2011)^51^ | PFS; defined to be the time interval *from enrollment in the study* to date of relapse |
| Zinzani et al (2011)^52^ | EFS; was defined as the interval *from the date of enrollment* to the first evidence of progression, disease relapse, death from any cause, treatment discontinuation due to any adverse event, or patient withdrawal. |
| Zhao et al (2007)^53^ | PFS; was defined as the *time from diagnosis* to first evidence of progression or relapse, or to disease related death. Data were censored at other causes of death or if the patients were free of progression/relapse at follow-up. |

**Supplemental Table 3** Meta-regression analyses

1. Prognostic

| **Subgroup** | **Reference** | **No of studies** | **HR of reference group** | ***P* value** |
| --- | --- | --- | --- | --- |
|  | **Control** |  |  |  |
| Study design | prospective | 6 | 3·17 (95%CI 2·13-4·73) | 0·9369 |
|  | retrospective | 12 |  |  |
| % DLBCL | 100% DLBCL | 15 | 2·84 (95% CI 2·29-3·52)  4·55 (95% CI 2·94-7·03) | 0·0577 |
|  | 80-99% DLBCL | 3 |  |  |
| Visual Criteria | Deauville | 11 | 3·21 (95% CI 1·51 -5·19) | 0·6997 |
|  | IHP | 3 |  |  |
| Scanner^*^ | PET/CT | 12 | 2·85 (95%CI 2·28-3·56)  4·39 (95% CI 3·15-6·10) | 0·0332 |
|  | PET/CT + standalone | 5 |  |  |
| Radiotherapy | yes | 9 | 2·88 (95%CI 2·13-3·89) | 0·4069 |
|  | unknown/no | 8/1 |  |  |
| ASCT upfront | yes | 2 | 4·96 (95% CI 2·40-10·21) |  |
|  | no | 5 |  | 0·2073 |
|  | unknown | 11 |  | 0·2126 |
| Blinded review | yes | 9 | 3·12 (95%CI 2·31-4·21) | 0·9653 |
|  | unknown/no | 8/1 |  |  |
| Outcome measure | PFS | 14 | 2·87 (95% CI 2·28-3·62) | 0·1495 |
|  | EFS | 4 |  |  |

1. Diagnostic

| **Subgroup** | **Reference** | **No of studies** | **Moderator** | **No of studies** | ***P* value** |
| --- | --- | --- | --- | --- | --- |
| Study design | prospective | 7 | retrospective | 12 | NS |
| % DLBCL | 100% DLBCL | 16 | 80-99% DLBCL | 3 | 0·0503  (accuracy) |
| Visual Criteria | Deauville | 12 | IHP | 3 | NS |
| Scanner^*^ | PET/CT | 12 | PET/CT + standalone | 6 | NS |
| Radiotherapy | yes | 10 | unknown/no | 9 | NS |
| ASCT upfront | yes | 2 | no | 5 | NS |
|  |  |  | unknown | 12 | NS |
| Blinded review | yes | 10 | unknown/no | 9 | NS |
| Outcome measure | PFS | 15 | EFS | 4 | NS |

NS= not significant

^*^No information about type of PET system for one study

**Supplemental Fig. 1** Funnel plot of studies investigating interim PET in DLBCL


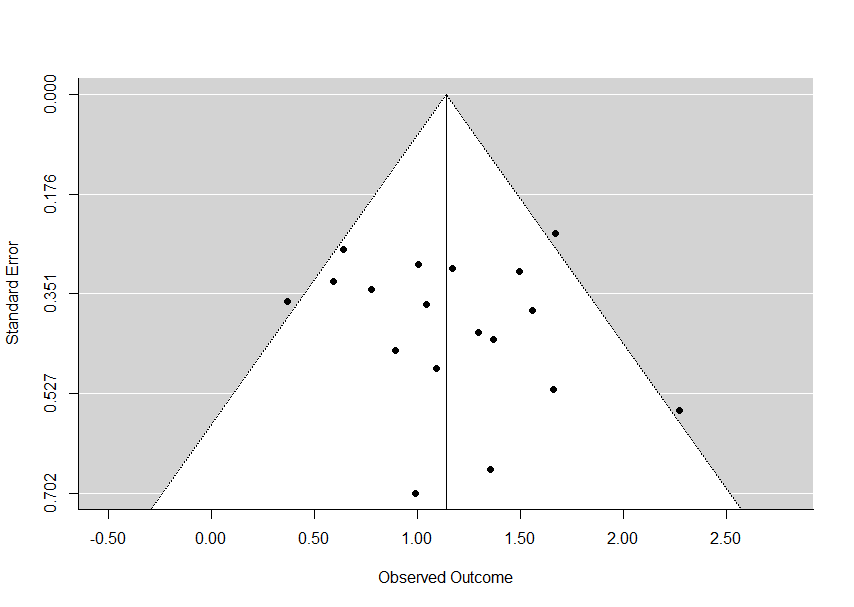


This plot shows the individual studies (black spheres) sorted by effect size (log HR) presented on the X-axis and standard error on the Y-axis. The solid vertical line corresponds to the estimated pooled log HR.

**Supplemental Fig. 2** Forest plots of sensitivity and specificity at two years of follow-up

**
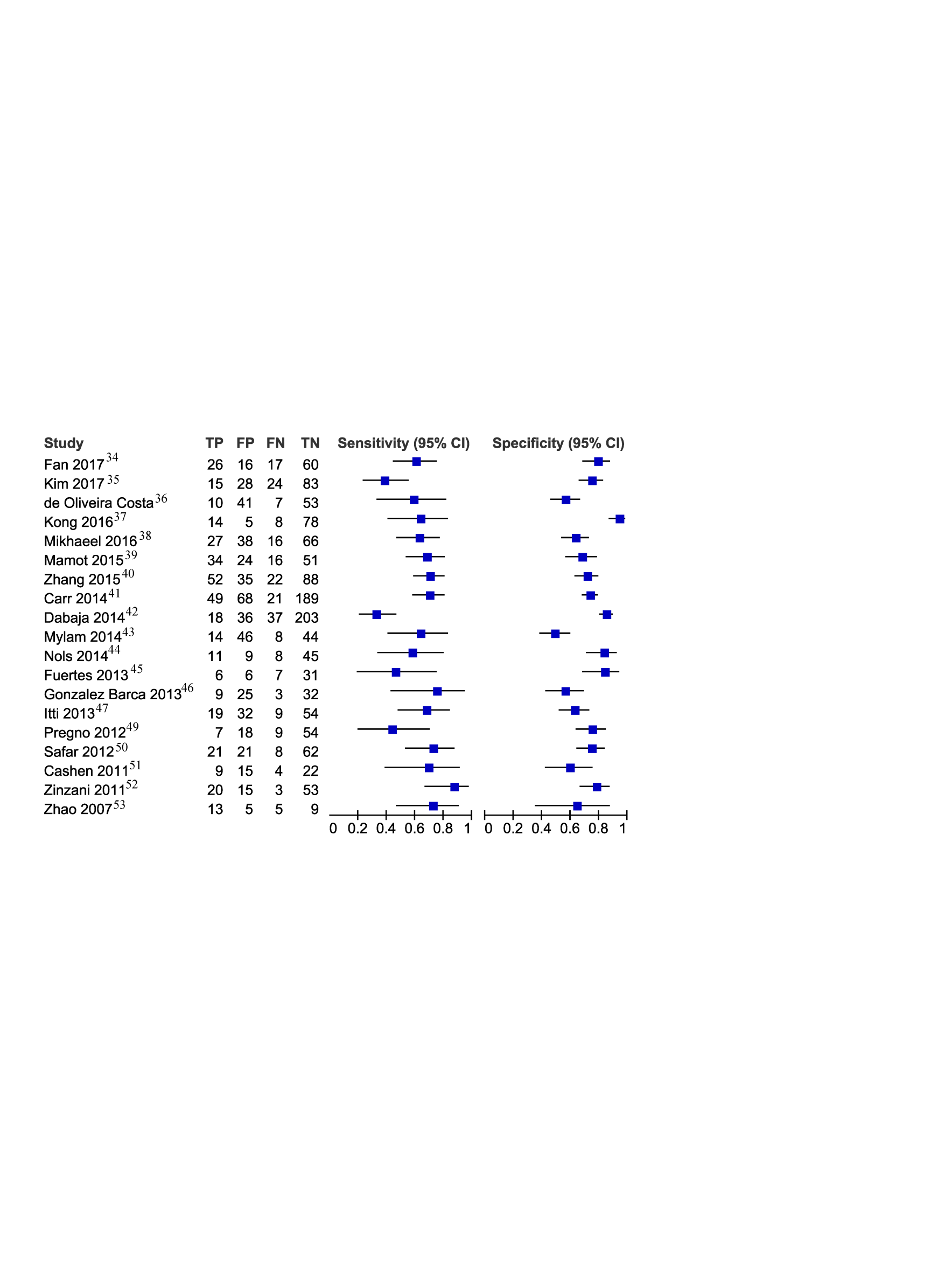
**

**Supplemental Fig. 3** Forest plot of univariate hazard ratios for interim PET scans after 2 cycles and assessed according to Deauville in diffuse large B-cell lymphoma.
